# CP-31398, a putative p53-stabilizing molecule tested in mammalian cells and in yeast for its effects on p53 transcriptional activity

**DOI:** 10.1186/1477-5751-3-5

**Published:** 2004-11-17

**Authors:** Stefan Tanner, Alcide Barberis

**Affiliations:** 1 ESBATech AG, Wagistrasse 21, CH-8952 Zürich-Schlieren, Switzerland

## Abstract

**Background:**

CP-31398 is a small molecule that has been reported to stabilize the DNA-binding core domain of the human tumor suppressor protein p53 *in vitro*. The compound was also reported to function as a potential anti-cancer drug by rescuing the DNA-binding activity and, consequently, the transcription activation function of mutant p53 protein in mammalian tissue culture cells and in mice.

**Results:**

We performed a series of gene expression experiments to test the activity of CP-31398 in yeast and in human cell cultures. With these cell-based assays, we were unable to detect any specific stimulation of mutant p53 activity by this compound. Concentrations of CP-31398 that were reported to be active in the published work were highly toxic to the human H1299 lung carcinoma and Saos-2 cell lines in our experiments.

**Conclusion:**

In our experiments, the small molecule CP-31398 was unable to reactivate mutant p53 protein. The results of our *in vivo *experiments are in agreement with the recently published biochemical analysis of CP-31398 showing that this molecule does not bind p53 as previously claimed, but intercalates into DNA.

## Background

The tumor suppressor protein p53 protects organisms from malignancy by either inducing programmed cell death or by arresting the cell cycle in response to cellular stress. The intracellular concentration of p53 is tightly regulated at the posttranslational level and the protein is very unstable under physiological conditions. Upon stress, p53 is stabilized and can act as a potent transcription factor that activates a plethora of downstream target genes [1,2]. The p53 target genes can be grouped into classes according to their effect on a cell. One class is represented by p21^CIP^, a cyclin dependent kinase inhibitor that is a potent inhibitor of the cell cycle. Another class of p53 target genes, of which bax is the most known representative, mediates p53-induced apoptosis. Other p53 target genes prevent the process of angiogenesis [2].

Not surprisingly, p53 is inactivated in a wide variety of human cancers [1,3]. Most mutations found in cancers are mis-sense mutations mapping to the central core domain of p53, which confers sequence-specific DNA binding activity to the protein. These mutations can cause destabilization of the core domain and loss of the DNA binding function. Thus, most mutant p53 proteins lack the ability to bind the DNA control elements of their target genes and fail to activate their expression. As a consequence, cells lacking functional p53 are unable to arrest the cell cycle or to undergo apoptosis in response to genotoxic stress. Since lack of p53 function plays such a central role in cancer development and in resistance to treatment, there has been much interest in the search of means and molecules to reactivate mutant forms of p53 [4-9].

A report by Foster et al. [7] generated special interest since it reported the discovery of a class of small molecules that was able to stabilize the p53 core domain. Not only were these compounds reported to stabilize the active conformation of wild type p53 but they were also shown to stabilize mutant p53 forms and enable them to activate transcription of p53 target genes. While the initial screening was conducted by an *in vitro *assay, activity of these compounds was subsequently confirmed in cell culture experiments and in a xenograft tumor mouse model [7]. One of their compounds, termed CP-31398, was reported to increase reporter gene activation by mutant p53 proteins about tenfold in the human p53-null lung carcinoma cell line H1299.

We tested CP-31398 in a yeast cell-based assay and in human tissue culture cells. We could not detect any reactivation of mutant p53 in these cellular assays. Our results are in agreement with, and provide support to the results obtained by Rippin et al. [10], which indicate that CP-31398 intercalates with DNA rather than binding p53.

## Results

**Figure 1:**
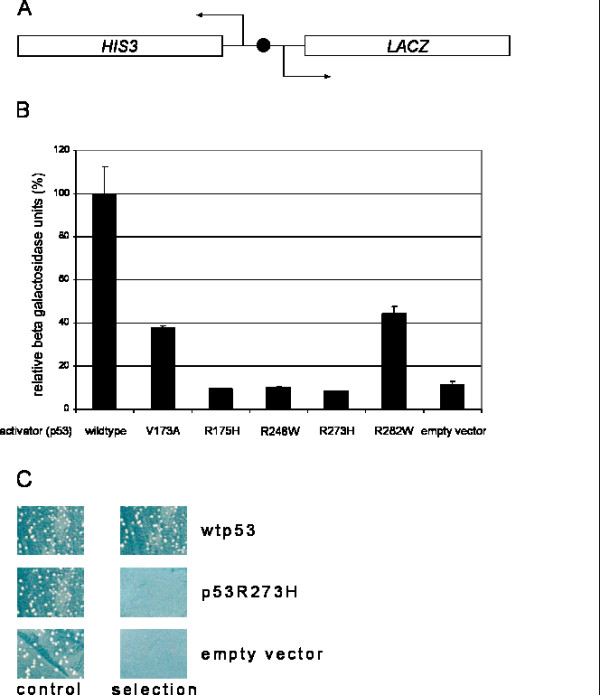
Human p53 protein activates transcription from a reporter construct in *Saccharomyces cerevisiae*. (A) Schematic representation of our yeast reporter construct integrated into our yeast strain. The black circle represents a single p53 responsive element from the human p21 promoter. (B) β-galactosidase assay to measure activation of the *lacZ *reporter gene. Wild type p53 and the indicated point mutant variants were transformed into the p53 responsive reporter strain and β-galactosidase activity in solution was determined. The activity of wild type p53 was arbitrarily set to 100%. p53R282W and p53V173A showed about 40% of activation compared to wild type p53. No activation of the reporter gene was detected in yeast cells containing the other point mutant variants. Average and standard deviation were determined from three independent experiments. (C) Growth on selective plates containing 20 mM 3-AT depends on expression of the *HIS3 *reporter gene and correlates with the activation of the *lacZ *reporter gene. Control plates consist of standard drop-out plates lacking the corresponding growth marker without 3-AT. Growth under selective conditions was dependent on activation of the p53 dependent reporter gene.

The yeast *Saccharomyces cerevisiae *does not contain p53 homologous proteins. However, it has been demonstrated that p53 expressed in yeast can function as a potent transcriptional activator of artificial genes bearing its specific recognition sequence [11]. To test different mutant forms of p53 and the potential effect of various molecules on the activity of such mutants, we constructed a yeast strain carrying an integrated bi-directional reporter gene construct in which a single p53 binding site from the human p21^CIP1 ^promoter [12] was inserted between the divergent *HIS3 *and *lacZ *genes (figure 1A). The p53-dependent expression of the yeast marker gene *HIS3 *allows growth selection on media lacking histidine and containing 3-amino-triazole (3-AT), which is a competitive inhibitor of the *HIS3 *gene product. The p53-dependent activation of this reporter gene is convenient for library screening, while expression of the bacterial *lacZ *gene allows verification and quantitation of the transcriptional activity of the various p53 forms and putative modulators.

Transformation of this strain with an episomal plasmid expressing human wild type p53 led to activation of the integrated *lacZ *and *HIS3 *reporter genes, which resulted in increased β-galactosidase activity (figure 1B) and cell growth on plates lacking histidine and containing 20 mM 3-AT (figure 1C). In contrast, expression of three mutant forms of p53 [1] with point mutations in their DNA-binding domain that completely abolish sequence-specific DNA-binding activity (p53R175H, p53R248W, p53R273H) did not activate transcription of the reporter genes (figure 1B and 1C, and data not shown). Expression of mutant forms that retain some DNA-binding activity *in vitro *and in mammalian cells [13] led to reduction of reporter gene expression compared to wild type p53 (figure 1B). All p53 variants were expressed to comparable levels, as verified by western blot analysis (data not shown).

Thus, the results of these transcriptional assays, taken together with published results of experiments performed in mammalian cells, indicate that the relative transcriptional activity of wild type p53 and the tested derivatives is comparable in yeast and in human cells.

Since lack of p53 function plays such a central role in cancer development and in resistance to chemotherapeutic treatment, many efforts have been directed towards trying to reactivate mutant forms of p53 [4-9,14]. The report by Foster et al. [7] generated special interest since it presented the discovery of a small molecule (CP-31398) that was able to stabilize the core domain of p53 *in vitro*. In addition, this compound was reported to enable some otherwise silent p53 mutants to activate transcription from target gene promoters in cell culture experiments.

**Figure 2:**
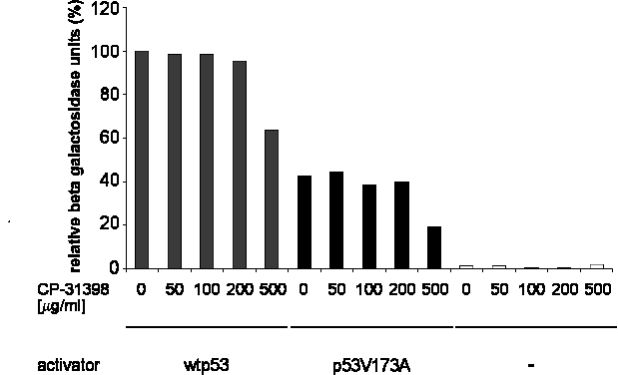
Treatment with the p53 stabilizing compound CP-31398 shows no effect on reporter gene activity in yeast. Yeast cells expressing wild type p53 (lanes 1–5), p53V173A (lanes 6–10) or empty vector (-, lanes 11–15, white bars) were incubated with the concentrations of CP-31398 indicated (0–500 μg/ml) and expression of β-galactosidase was determined. β-galactosidase activity of wild type p53 without CP-31398 treatment was arbitrarily set to 100%. Yeast cells were treated with CP-31398 for 16 hours.

We tested the effect of CP-31398 on human p53 activity in our p53-responsive yeast strain. Yeast cells expressing either wild type p53 or the mutant p53R173A were grown in media containing increasing concentrations of CP-31398. Activation of transcription of the p53-dependent reporter gene was assessed by measuring β-galactosidase activity in extracts from these cells (figure 2). No significant difference in *lacZ *reporter gene expression was observed between untreated cells and cells that were incubated with increasing concentrations of the compound. Very high concentrations of CP-31398 (500 μg/ml) reduced reporter gene activity, both in the case of wild type p53 expression and in the case of p5R173A expression. Results of growth assays on selective plates to indirectly measure *HIS3 *expression paralleled our data from the *lacZ *experiments (data not shown).

**Figure 3:**
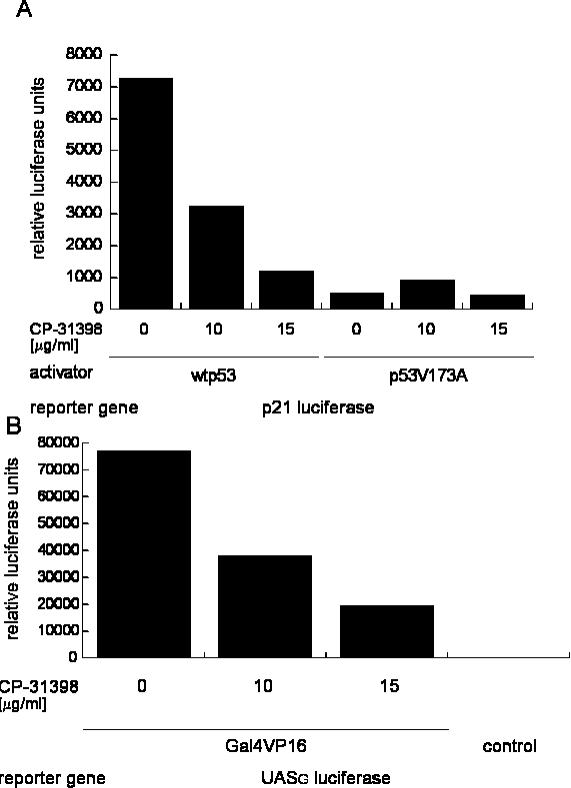
Treatment of H1299 lung carcinoma cells with CP-31398 provokes massive cell death and p53 independent decline of luciferase reporter gene activity. (A) H1299 cells were transfected with expression constructs for wild type p53 (lanes 1–3) and p53V173A (lanes 4–6). All the samples were cotransfected with a p53-responsive luciferase reporter (p21 luciferase, containing a single p53 responsive p53 binding site from the human p21 promoter, termed WWP-luc, see material and methods) and a constitutive reference β-galactosidase construct (CMV-*lacZ*) for normalization. These cells were subsequently incubated with 0, 10, 15 μg/ml CP-31398 respectively and relative luciferase activities were determined. (B) H1299 cells were transfected with an expression construct for the synthetic activator *GAL4*-VP16. All samples were cotransfected with a gal4p responsive luciferase reporter (UAS_G _luciferase) and a reference β-galactosidase plasmid (CMV-*lacZ*) for normalization. The control cells were transfected with CMV-*lacZ *and UAS_G _luciferase only. These cells were subsequently incubated with 0, 10 and 15 μg/ml CP-31398 and relative luciferase activities were determined. The cells were treated with CP-31398 for 16 hours.

Since these negative results regarding the lack of expected effects of CP-31398 on p53 could be due to our assay system in yeast, we tested CP-31398 in experiments with human tissue culture cells. We transfected the human p53-null H1299 lung carcinoma cell line that was also used for some of the experiments described by Foster et al. [7] with plasmid DNA expressing either human wild type p53 or the p53R173A mutant together with a reporter plasmid carrying a p53-responsive luciferase gene [12]. When we treated these cells with CP-31398 in concentrations that were shown to be effective by Foster et al. (5–20 μg/ml and higher concentrations), reporter gene signals decreased and massive cell death was observed (figure 3A and data not shown). Lower concentrations that showed no obvious toxicity to the cells had no significant effect on reporter gene activity. We observed very similar effects when we performed corresponding experiments in the osteosarcoma cell line Saos-2 (p53 null cell line) (data not shown).

Cell death and decreased reporter gene activity was not dependent on the expression of p53 since treatment with CP-31398 of the same cell lines expressing the unrelated activator GAL4-VP16 co-transfected with the respective reporter construct caused similar toxicity and lower reporter gene activity (figure 3B).

**Figure 4:**
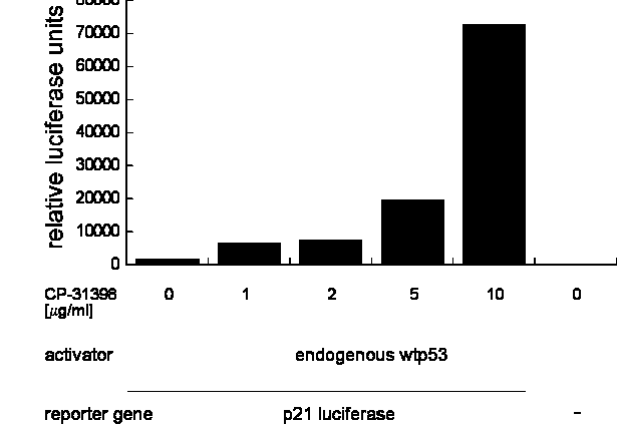
Treatment of HeLa cervical carcinoma cells with CP-31398 leads to p53 dependent induction of the luciferase reporter. HeLa cells were transfected with a p53 responsive reporter gene (WWP-luc) and a reference β-galactosidase plasmid (CMV-*lacZ*) for normalization. Control cells were transfected with CMV-*lacZ *alone. The cells were subsequently incubated with CP-31398 (0–10 μg/ml) and relative luciferase activities were determined. Cells were treated for 16 hours.

We next tested whether CP-31398 might have an effect on endogenous wild type p53 in the human cell line HeLa. These cells express wild type p53 protein, but p53 levels are low because of the presence of the viral HPV E6 protein, which targets p53 for degradation [15]. We transfected HeLa cells with the same p53-dependent luciferase reporter construct that was used with the other cell lines and treated the cells with increasing concentrations of CP-31398 (figure 4). To our surprise, there was a strong increase in reporter gene activation. When we expressed additional human wild type p53 from a transfection plasmid, the signal increased even more (data not shown). In contrast to the previous effect on other cell lines described above, we did not observe any significant cell death in the case of HeLa.

**Figure 5:**
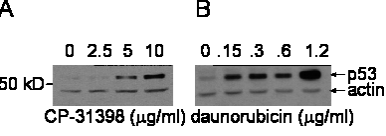
Western Blot analysis of HeLa cells treated with CP-31398 and daunorubicin. (A) HeLa cells were treated with increasing concentrations of CP-31398 and protein extracts were subjected to SDS-PAGE and subsequent detection with an anti-p53 antibody (DO-1). (B) HeLa cells were treated with the established p53 inducing agent daunorubicin. Protein extracts were subjected to SDS-PAGE and subsequent detection with an anti-p53 antibody (DO-1). Expression of actin is detected as a loading control in experiments 5A and B.

We subjected extracts from HeLa cells treated with CP-31398 to western blot analysis. The p53 signals correlated with increasing CP-31398 concentrations, whereas the actin control signals did not (figure 5A). These results are consistent with a classical response to genotoxic stress by compounds causing stabilization of p53 [16].

We also measured changes in p53 levels in HeLa cells after treatment with increasing concentration of daunorubicin, a known anticancer agent that is highly cytotoxic by a number of proposed mechanisms – intercalation into DNA among them [17]. We found, as expected, that daunorubicin treatment led to a progressive stabilization of p53 in HeLa cells comparable to the response when cells were treated with CP-31398 (figure 5B).

## Discussion

We assessed the proposed p53 stabilizing action of CP-31398 in yeast cells and in human cells. CP-31398, a compound isolated in an antibody-based *in vitro *screen, was reported to stabilize the p53 DNA-binding core domain and to reactivate mutant p53 *in vivo *[7]. We were unable to detect any effect of CP-31398 on p53-dependent reporter gene activation by a mutant form of human p53 neither in human cells nor in yeast cells. In our hands, CP-31398 did not stabilize mutant p53 proteins so as to show differences in activation of p53-dependent reporter genes in yeast and in mammalian cells. In addition, concentrations that were shown to be effective in cell culture by Foster et al. [7] led to extensive cell death. Most importantly, such cell death was independent of p53 expression.

The p53 protein expressed within yeast cells functions as a potent transcriptional activator. Reconstitution of transcriptional activation by p53 in a heterologous, yet cellular system such as a yeast cell should be suitable to assess DNA-binding and transcriptional activation activity regardless of posttranslational modifications and other influences that are inevitable when p53 is studied in the context of its regulatory network in mammalian cells. It has been proposed that such posttranslational modifications like acetylation and phosphorylation activate the latent DNA binding activity of p53 by allosteric mechanisms [18]. However, more recent *in vivo *and *in vitro *studies question whether DNA binding itself is regulated at all and suggest that induction of p53 activity primarily occurs at the level of increasing protein concentration within the nucleus [16,19,20]. The evident p53 activity in yeast cells, in which the proposed mammalian-specific p53 modifying enzymes are missing, seems to be more readily consistent with the conclusions of such studies. With our system in yeast, we should be able to detect stabilization of the p53 core domain as long as this leads to increased binding of p53 to its specific DNA recognition sequence and subsequent activation of reporter gene expression. Therefore, our yeast system provides a convenient means to screen compound libraries for identifying molecules that can reactivate mutant p53 proteins in a cellular environment. Thanks to the easy genetic malleability of yeast and the lack of endogenous p53-related pathways, cellular screens with this organism should allow not only identification of compounds that can permeate cellular membranes and be active in an intracellular environment but also rapid exclusion of molecules that are not specific for the chosen target.

In contrast to the results obtained with the exogenous expression of wild type p53 in yeast cells or with the H1299 and Saos-2 human cells, we observed a strong increase in wild type p53-dependent reporter gene activation in HeLa cells. These cells showed no apparent cell death after treatment with CP-31398. Wang et al. [21] reported stabilization of wild type p53 and an increase in p53 levels in other cell lines. These observations are consistent with the results we obtained in HeLa cells. These authors also reported that ubiquitination and degradation of wild type p53 is blocked by CP-31398. This effect seems to be specific to mdm2-mediated p53 degradation since HPV (human papilloma virus) E6-mediated degradation of p53 was unaffected. We do not know why we do not see any stabilization of exogenous p53 in H1299 or Saos-2 cells, but it is possible that unspecific toxicity induced by CP-31398 masks the increasing p53-dependent reporter signal. While these results indicate that CP-31398 might stabilize wild type p53, they do not explain the mechanism. Direct interaction and stabilization of p53 is not excluded. However, other explanations seem plausible. Stabilization of the core domain structure by CP-31398 as proposed in the original article should presumably have no effect on p53 protein levels. But p53 levels increase after treatment with CP-31398. Such a response is in line with a classical stabilisation of p53 after genotoxic stress. In contrast, Wang et al. reported that no serine 15 or 20 phosphorylation was detected in their cells after treatment with CP-31398. Interaction with mdm2 was unaffected, but p53 degradation was nevertheless blocked [21]. Therefore, it remains unclear by which mechanism CP-31398 stabilizes p53; it seems unlikely that core domain stability and DNA binding are influenced by CP-31398 directly. It is interesting to note that CP-31398 can intercalate into DNA as reported by Rippin et al. [10]. This intercalation is probably toxic to the cell and likely induces a classical p53 response, similar to the known p53 inducer daunorubicin.

Our results strongly suggest a classical p53 stabilization through reduced degradation due to genotoxic effects caused by CP-31398. In fact, wild type p53 levels changed quite dramatically in HeLa cells, which are resitant to the apoptotic effects of p53, whereas the other human cell lines did not survive the treatment, probably because they underwent apoptosis in response to CP-31398 [22]. In support to this interpretation, our control substance daunorubicin showed very similar and expected results as those obtained with CP-31398.

## Conclusions

In contrast to the results reported by Foster et al. [7], we did not detect any stimulation of mutant p53 activity *in vivo *by CP-31398, a potential anti-cancer compound. Concentrations of CP-31398 that were reported to be active in the published work were highly toxic to human cells in our experiments. The results of our *in vivo *experiments are in agreement with the recently published biochemical analysis of CP-31398, which shows that this molecule does not bind p53 as previously claimed, but rather intercalates into DNA.

## Methods

### Yeast strains

The yeast strain used in our experiments is a derivative of the *S. cerevisiae *strain JPY5 [23] (*MAT**ura3-52 his3Δ200 leu2Δ1 trp1Δ63 lys2Δ385*). The p53 responsive yeast strain was constructed by integration of the reporter construct described in the result section and in figure 1A into the *HIS3 *locus by homologous recombination. The integrating p21 reporter plasmid was linearized with *AflII *that cuts in the 3' untranslated region (3'UTR) of the *S. cerevisiae HIS3 *gene.

### Yeast growth and manipulations

Yeast genetic techniques and media were as described in [24]. For selection of plasmids, dropout media containing all except the specified amino acids were used. Yeast transformation was performed by the lithium acetate procedure [25].

### Recombinant plasmids

All p53 forms tested in yeast were expressed from the vector pGAD424 (Clontech, Inc). Wild type p53 was subcloned from a mammalian expression vector with primers containing *HinDIII *restriction sites by polymerase chain reaction (PCR). The PCR product was introduced into the *HinDIII *sites of pGAD424, removing the *GAL4*AD ORF from pGAD424. All the point mutant p53 variants were generated by assembled PCR with mismatched primer pairs and subsequent cloning into pGAD424 analogous to wild type p53. The yeast reporter plasmid was derived from pDE96 (yeast integrating plasmid, bi-directional *HIS3*, *lacZ*) [26] by introduction of a hybridised double stranded oligo containing the p53 responsive element from the p21^CIP1 ^promoter (p21_sense_SalI 5'-TCG AGC CGT CAG GAA CAT GTC CCA ACA TGT TGA GCT G-3' and p21_anti_XbaI 5'-CTA GCA GCT CAA CAT GTT GGG ACA TGT TCC TGA CGG C-3') into the *XbaI *and *SalI *sites of the vector backbone. The plasmid WWP-luc is described in [12].

The mammalian p53 expression plasmids were constructed by subcloning the *HinDIII *p53 fragments from the yeast expression vectors into the *GAL4 *expression plasmid pSCETV-*GAL4*(1-93)RV, this resulted in p53 expression under the control of the CMV promoter. The mammalian GAL4 dependent reporter Gal5-luc contains five GAL4 responsive binding sites in front of the luciferase cassette [27]. Gal4-VP16 is described elsewhere [28].

### Yeast β-galactosidase assay

Yeast β-galactosidase assays in solution using permeabilized cells were performed as described in [24]. Activity was normalized to the number of cells assayed.

### Mammalian cell culture

Cells were obtained from ATCC (American Type Culture Collection, Manassas, Virginia, USA) and cultured according to the recommendations of ATCC.

### Transient transfection and luciferase assays

We used Polyfect^® ^transfection reagent (Qiagen, Inc) according to manufacturers recommendations for transfection of all cell lines. Cells for luciferase assays and western blotting were harvested by scraping 48 hours after transfection and subjected to three freeze thaw cycles in 100 mM potassium phosphate pH 7.8 1 mM dithiothreitol buffer. Supernatants were clarified by centrifugation (5 min, 13000 rpm) and resuspended in 100 μl extraction buffer. 10 μl of extract was mixed with 100 μl luciferase assay solution (Promega) and analyzed in a luminometer (EG&G Berthold Lumat LB 9507). β-galactosidase assays were performed according to standard methods using 50 μl of the extract and luciferase units were normalized according to β-galactosidase values. All measurements were performed from at least two independent transfections experiments.

### Western blot analysis and antibodies

Protein extracts were prepared as described above. Proteins were separated by SDS-PAGE, electrophoretically transferred to nitrocellulose membranes, and western blotting was performed according to standard procedures. Anti-p53 antibody DO-1 (Santa Cruz Biotechnology, Inc) reacts with an amino terminal epitope mapping between amino acid residues 11–25 of wild type and mutant p53. Anti-actin antibody (I-19; Santa Cruz Biotechnology, Inc) is an affinity purified goat polyclonal antibody raised against a peptide mapping to the carboxy terminus of human actin.

## Authors' contributions

All experimental work was carried out by ST. AB conceived of the study and participated in its design and coordination. Both authors read and approved the final manuscript.
